# Contribution of cerebrospinal fluid antibody titers and sex to acute cerebral blood flow in patients with anti-NMDAR autoimmune encephalitis

**DOI:** 10.3389/fimmu.2024.1299898

**Published:** 2024-03-01

**Authors:** Ailiang Miao, Kai Wang

**Affiliations:** ^1^Department of Neurology, The First Affiliated Hospital of Anhui Medical University, Hefei, China; ^2^Department of Neurology, The Affiliated Brain Hospital of Nanjing Medical University, Jiangsu, Nanjing, China; ^3^School of Mental Health and Psychological Sciences, Anhui Medical University, Hefei, China; ^4^Institute of Artificial Intelligence, Hefei Comprehensive National Science Center, Hefei, China; ^5^Anhui Province Key Laboratory of Cognition and Neuropsychiatric Disorders, Hefei, China; ^6^Collaborative Innovation Center of Neuropsychiatric Disorders and Mental Health, Hefei, China; ^7^Anhui Provincial Institute of Translational Medicine, Anhui Medical University, Hefei, China

**Keywords:** anti-NMDAR autoimmune encephalitis, arterial spin labeling, cerebral blood flow, CSF antibody titers, sex

## Abstract

**Objective:**

The objective of this study was to elucidate the contribution of cerebrospinal fluid (CSF) antibody titers (AT) and sex to acute cerebral blood flow (CBF) in patients diagnosed with anti-*N*-methyl-d-aspartate receptor autoimmune encephalitis (NMDAR AE).

**Methods:**

Forty-five patients diagnosed with NMDAR AE were recruited from December 2016 to January 2023. The acute CBF in patients with NMDAR AE at the early stage of the disease was analyzed using arterial spin labeling. The groups were compared based on CSF AT and sex. The connectivity of the CBF in the region of interest was also compared between groups.

**Results:**

The patients with different CSF AT exhibited varied brain regions with CBF abnormalities compared to the healthy subjects (*p* = 0.001, cluster-level FWE corrected). High antibody titers (HAT) in CSF contributed to more brain regions with CBF alterations in female patients than in female patients with low antibody titers (LAT) in CSF (*p* = 0.001, cluster-level FWE corrected). Female patients with HAT in CSF displayed more decreased CBF in the left post cingulum gyrus, left precuneus, left calcarine, and left middle cingulum gyrus than the male patients with the same AT in CSF (*p* = 0.001, cluster-level FWE corrected). All patients with NMDAR AE showed increased CBF in the left putamen (Putamen_L) and left amygdala (Amygdala_L) and decreased CBF in the right precuneus (Precuneus_R), which suggests that these are diagnostic CBF markers for NMDAR AE.

**Conclusion:**

CSF AT and sex contributed to CBF abnormalities in the patients diagnosed with NMDAR AE. Altered CBF might potentially serve as the diagnostic marker for NMDAR AE.

## 1 Introduction

Anti-*N*-methyl-d-aspartate receptor autoimmune encephalitis (NMDAR AE) is a common type of AE associated with antibodies against NMDARs in cerebrospinal fluid (CSF) (1, 2). Patients and their families suffered from heavy financial and mental burdens due to NMDAR AE. Prompt and accurate diagnosis, followed by timely treatment, are crucial factors in enhancing the prognosis of NMDAR AE (3). However, the clinical diagnosis of this disease mainly depends on the antibody detection in serum and CSF (4), which takes at least 3 days in China. Nonspecific findings of conventional MRI could not contribute to the diagnosis of NMDAR AE (5–7). In our previous study, obvious cerebral blood flow (CBF) alterations detected by pseudocontinuous arterial spin labeling (pcASL) were observed in patients with NMDAR AE (5). In the previous case reports, markedly increased CBF was observed in the patients suffering from NMDAR AE, suggesting that evaluating CBF could assist in the early diagnosis of the disease (8, 9). In our previous study, CSF antibody titers (AT) and sex were associated with clinical symptoms and electroencephalography background activity. AT in CSF were associated with poor outcomes (7). Thus, the aim of the current study was to clarify the contribution of different CSF antibody titers and sexes to CBF, and to search for CBF markers in patients with NMDAR AE.

## 2 Materials and methods

### 2.1 Participants

A total number of 45 patients with acute CBF and 65 healthy subjects from the Department of Neurology of First Affiliated Hospital of Anhui Medical University and Affiliated Brain Hospital of Nanjing Medical University were recruited from December 2016 to January 2023. The inclusion criteria of NMDAR AE were as follows (4): (1) patients with positive CSF NMDAR antibody in patients; and (2) patients with rapid development (within 3 months) of various symptoms such as mental behavioral disorders, seizures, movement disorders, cognitive dysfunction, speech disorder, disturbance of consciousness, or central hypoventilation. If the patients were diagnosed with other conditions, such as viral encephalitis, brain tumor, metabolic diseases, and drug poisoning, they would be excluded.

The collection of data included neurological symptoms, MRI findings, the EEG background activity, modified Rankin scale (mRS) scores, as well as age and sex. The abnormal electroencephalography (EEG) background activity was classified mild diffuse slowing (DS), moderate DS, and severe DS (7, 10, 11). The initial stage was defined as the period within 14 days following the onset of symptoms (6, 12). The peak stage was defined as the period occurring between 14 and 60 days after symptom onset (6, 12). According to our previous studies and our clinical observation, anti-NMDAR AT of 1:1 and 1:3.2 in CSF were considered as low antibody titers (LAT), while anti-NMDAR AT of 1:10, 1:32, 1:100, and 1:1,000 in CSF were defined as high antibody titers (HAT) (7, 10, 13). During the acute stage of the disease, antibodies in CSF were measured using indirect immunofluorescence technique by cell-based assays (Euroimmun kits, Germany).

### 2.2 MRI data

The MRI data were acquired using a 3.0-T MR system manufactured by General Electric in the United States. The utilization of earplugs served to minimize scanner noise, while the application of firm yet cozy foam padding was employed to mitigate head movement. Resting-state brain perfusion imaging and sagittal 3D T1-weighted images were obtained using a pcASL sequence with a 3D fast spin-echo acquisition and brain volume sequence, respectively. During the MRI scans, all participants were given instructions to close their eyes, maintain physical relaxation, minimize movement, refrain from engaging in specific cognitive activities, and avoid falling asleep.

### 2.3 CBF calculation

The control maps were subtracted by the label maps to generate ASL differences maps (5, 14, 15). The CBF images were calculated by averaging the three ASL difference images in conjunction with the proton-density-weighted reference images. The CBF maps were obtained from the ASL difference maps. The CBF maps were normalized to a PET-perfusion template in the Montreal Neurological Institute space using SPM12 software to create the normalized CBF maps. Then, the standardized CBF images were created by dividing the normalized CBF maps of each voxel by the mean CBF of the whole brain. The smoothed images were created through smoothing the standardized CBF maps with a Gaussian kernel of 8 mm × 8 mm × 8 mm full width at half maximum (FWHM).

### 2.4 Normalized CBF analyses

The CSF antibody titers and sex served as grouping criteria. The differences of CBF among multiple groups were compared using analysis of variance with age and sex as the nuisance, and a spatial mask was created. Following that, the independent-samples *t*-test was employed to compare the variations in CBF among different groups within the spatial mask generated earlier. Additionally, age and sex were taken into account as covariates. To control for multiple comparisons (MC), the cluster-level family-wise error (FWE) method was employed with an adjusted significance threshold of *p* = 0.001. The clusters exhibiting a significant disparity among groups were utilized for illustration.

### 2.5 CBF connectivity analyses

The assessment of CBF connectivity among specific brain areas can be achieved by analyzing the correlation coefficient of concurrent changes in CBF among brain regions. The specific procedures are enumerated as follows: (1) Brain regions with CBF alteration in all patients with different AT were selected as the seed region of interest (ROI). (2) The dpabi software was used to extract CBF measurement of each ROI for each subject from their respective CBF maps. The connectivity of CBF between each ROI and all other global voxels across individuals was computed using a single-sample *t*-test for each group, while controlling for confounding variables such as sex and age. The statistical analyses were performed to recognize the voxels exhibiting positive or negative correlations with the CBF value of each seed ROI within each group. The MC were adjusted using the cluster-level FWE method (*p* = 0.001). (3) For each seed ROI, the spatial mask was created using the connectivity images of CBF between the two groups, where the CBF values of each voxel were associated with those of the ROI in either group. (4) The connectivity of CBF might exhibit distinct slopes between the two groups for any given pair of voxels, indicating variations in connectivity of CBF across the groups. The voxels expressing a meaningfully different connectivity of CBF with each ROI between healthy subjects and patients with NMDAR AE were mapped by establishing specific two-sample *t*-test within the connectivity spatial mask generated in the previous step, while controlling for age and sex. The MC were adjusted using the cluster-level FWE method (*p* = 0.001). (5) The dpabi software was utilized to extract CBF measurement of each seed ROI from an individual CBF image. The correlation between ROIs was calculated by the Pearson correlation (*p* < 0.05) (5, 16, 17).

## 3 Results

We retrospectively identified 45 patients with acute CBF within a mean 14.7 days of symptom onset from 5 December 2016 to 16 March 2023 (25 female patients; 20 male patients). HAT in CSF was detected in both female (*n* = 15) and male (*n* = 9) patients with NMDAR AE. Similarly, LAT in CSF was found in female patients (*n* = 10) as well as male patients (*n* = 11). Behavioral changes were observed in 51.1% (23/45) of patients. Seizures were observed in 48.9% (22/45) of the patients. The clinical manifestations were more pronounced in female patients with HAT in CSF compared to those with LAT in CSF (mean: 3.20 ± 1.74 *vs.* 1.4 ± 0.52, *p* = 0.004). Normal T2/Flair and focal high cerebral blood flow (HCBF) were observed in 57.8% (26/45) of the patients with NMDAR AE. Abnormal T2/Flair were observed in 20% of patients (9/45), showing HCBF. The level of EEG background activity exhibited a higher severity in female patients with HAT in CSF as opposed to those with LAT in CSF (Mann‒Whitney *U* test, *p* = 0.001). The features of the individuals diagnosed with NMDAR AE are displayed in **Table 1**.

**Table 1:** Clinical characteristics of patients with NMDAR AE.

Clinical features	Patients	Mean ± standard deviation	*p*-Value
**Sex** **Female** **^H^Female** **^L^Female** **Male** **^H^Male** **^L^Male**	25 15 10 20 9 11		
**Age** **^H^Female_age** **^L^Female_age** **^H^Male_age** **^L^Male_age**		25.8 ± 11.39 18.2 ± 5.25 28.44 ± 4.93 28.18 ± 13.00	
Symptom presentation			
**Behavioral changes** **^H^Female** **^L^Female** **^H^Male** **^L^Male**	23 12 (12OS) 3 (3OS) 4 (2OS) 5 (3OS)		
**Seizures** **^H^Female** **^L^Female** **^H^Male** **^L^Male**	22 12 (3OS) 7 (6OS) 7 (4OS) 8 (8OS)		
**Disturbance of consciousness**	6		
**Cognitive dysfunction**	15		
**Speech disorder**	4		
**Focal limb weakness**	1		
**Movement disorders (involuntary movement)**	6		
**Number of symptoms** **Female^H^ *^vs^* ^L^** **Male^H^ *^vs^* ^L^**		3.20 ± 1.74 *vs.* 1.4 ± 0.52 1.78 ± 1.09 *vs.* 1.36 ± 0.50	*p* = 0.004 *p* = 0.28
**Total ASL**	45		
**Focal HCBF and abnormal T2/Flair**	9		
**Focal HCBF and normal T2/Flair**	26		
**Normal blood flow and T2/Flair**	10		
**EEG during peak stage** **Background activity**	39		
**^H^Female**	8 SDS; 5 moderate DS; 2 mild DS; 7 EDB		
**^L^Female**	2 moderate DS; 5 mild DS; 2 normal	^H^Female *vs.* ^L^Female	*p* = 0.001
**^H^Male**	5 mild DS		
**^L^Male**	1 moderate DS; 8 mild DS; 1 normal		
Therapy			
**First-line alone** **First-line and second-line**	45 12		
**mRS** **(follow-up from 9 to 81 months)** **0–1** **≥2**	Median 52 35 10 (2 died)		

HCBF, high cerebral blood flow; mild DS, mild diffuse slowing; moderate DS, moderate diffuse slowing; SDS, severe diffuse slowing; EDB, extreme delta brush; ASL, arterial spin labeling. H, high antibody titer in CSF (1:10, 1:32, 1:100, and 1:1,000). L, low antibody titer in CSF (1:1 or 1:3.2). OS, onset denotes the number of patients with the clinical symptoms manifesting onset symptoms.

### 3.1 CBF chances in female patients

**Figure 1:**
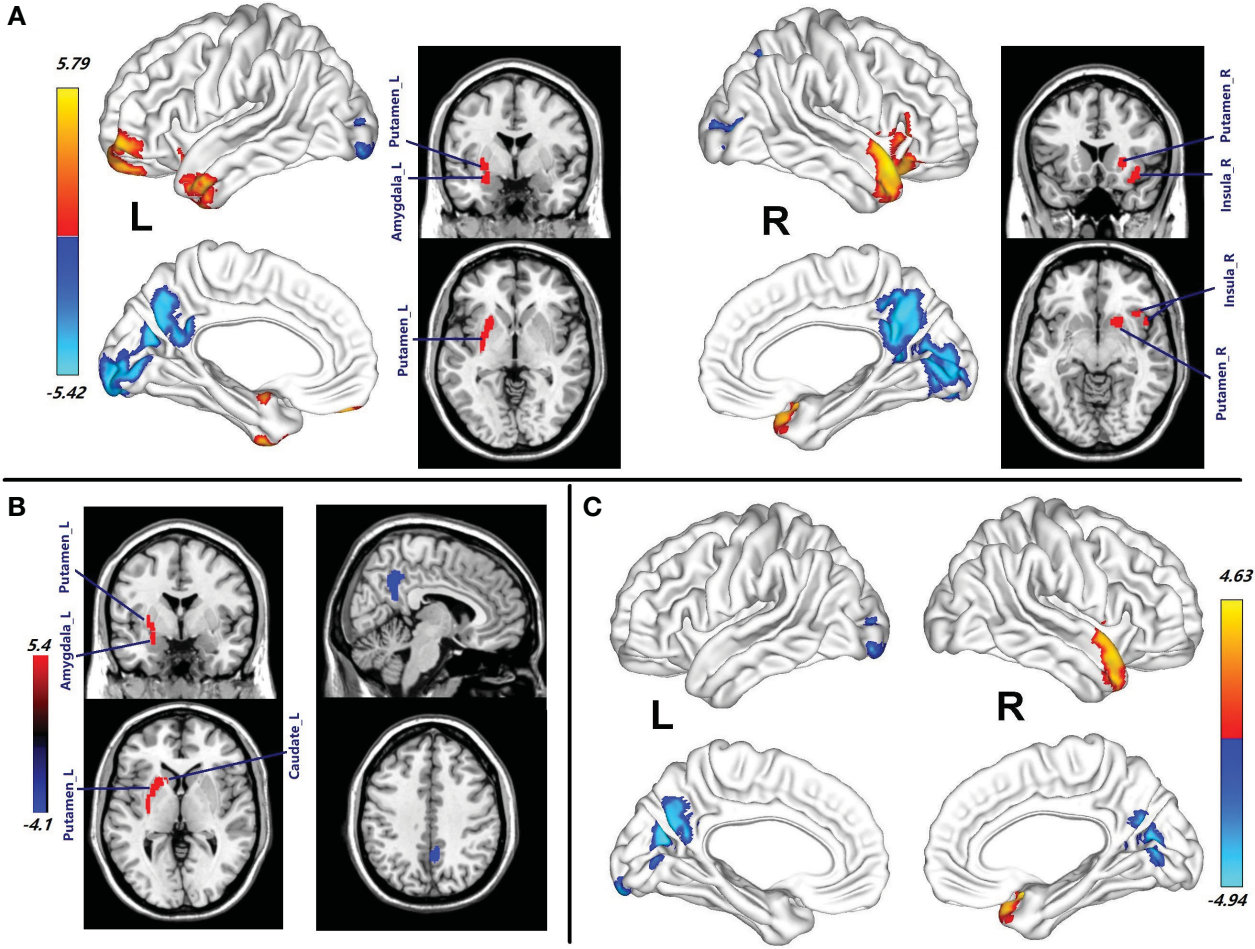
**(A)** Brain regions with CBF abnormalities between the female patients with HAT in CSF and female healthy subjects (*p* = 0.001, cluster-level FWE corrected). **(B)** Compared with the healthy female subjects, the brain regions with CBF alteration in the female patients with LAT in CSF (*p* = 0.001, cluster-level FWE corrected). **(C)** Different CBF abnormalities between the female patients with HAT and the female patients with LAT in CSF (*p* = 0.001, cluster-level FWE corrected). Cool color indicates the decrease in CBF of the patients with NMDAR AE. Warm color indicates the increase in CBF of the patients with NMDAR AE. HAT, high antibody titers; LAT, low antibody titers; NMDAR AE, anti-N-methyl-d-aspartate receptor autoimmune encephalitis.

The chances in CBF between the patients with HAT in CSF and the healthy female subjects are displayed in **Figure 1A** and **Table 2**. Female patients with HAT in CSF exhibited increased CBF in the left middle temporal pole (MTP_L), left inferior temporal gyrus (ITG_L), left middle temporal gyrus (MTG_L), left putamen (Putamen_L), left amygdala (Amygdala_L), left middle orbitofrontal gyrus (MOFG_L), left superior orbitofrontal gyrus (SOFG_L), left inferior orbitofrontal gyrus (IOFG_L), right superior temporal pole (STP_R), right IOFG (IOFG_R), right putamen (Putamen_R), right MTP (MTP_R), right insula (Insula_R), left triangle inferior frontal gyrus (TIFG_L), right caudate (Caudate_R), right olfactory (Olfactory_R), and right operculum inferior frontal gyrus (OIFG_R) (*p* = 0.001, cluster-level FWE corrected) (**Figure 1A**; **Table 2**). In contrast, these patients displayed meaningfully decreased CBF in the right precuneus (Precuneus_R), right calcarine (Calcarine_R), left calcarine (Calcarine_L), right lingual (Lingual_R), left precuneus (Precuneus_L), left lingual (Lingual_L), left middle occipital gyrus (MOG_L), left inferior occipital gyrus (IOG_L), right IOG (IOG_R), left cuneus (Cuneus_L), right cuneus (Cuneus_R), right fusiform (Fusiform_R), left post cingulum gyrus (PCG_L), right MOG (MOG_R), right middle cingulum gyrus (MCG_R), left superior occipital gyrus (SOG_L), right PCG (PCG_R), and right SOG (SOG_R) (*p* = 0.001, cluster-level FWE corrected) (**Figure 1A**; **Table 2**).

**Table 2:** Brain region CBF with significant group differences in patients.

Regions	Coordinates in MNI (*x, y, z*)	Cluster-level FWE	Peak *t* values	Cluster size (voxels)
Female patients with HAT in CSF >Healthy subjects				
**MTP_L**	−34, 0, −48	*p* = 0.001	5.01	91
**ITG_L**	−34, 0, −48		5.01	77
**MTG_L**	−34, 0, −48		5.01	71
**Putamen_L**	−28, 0, −6	*p* = 0.001	4.42	362
**Amygdala_L**	−28, 0, −6		4.42	35
**MOFG_L**	−40, 56, −8	*p* = 0.001	4.90	143
**SOFG_L**	−40, 56, −8		4.90	71
**IOFG_L**	−40, 56, −8		4.90	37
**STP_R**	54, 16, −10	*p* = 0.001	5.79	706
**IOFG_R**	54, 16, −10		5.79	327
**Putamen_R**	54, 16, −10		5.79	273
**MTP_R**	54, 16, −10		5.79	251
**Insula_R**	54, 16, −10		5.79	216
**TIFG_R**	54, 16, −10		5.79	59
**Caudate_R**	54, 16, −10		5.79	39
**Olfactory_R**	54, 16, −10		5.79	34
**OIFG_R**	54, 16, −10		5.79	32
Female patients with HAT in CSF < Healthy subjects				
**Precuneus_R**	26, −86, −6	*p* = 0.001	−5.42	817
**Calcarine_R**	26, −86, −6		−5.42	606
**Calcarine_L**	26, −86, −6		−5.42	577
**Lingual_R**	26, −86, −6		−5.42	454
**Precuneus_L**	26, −86, −6		−5.42	443
**Lingual_L**	26, −86, −6		−5.42	189
**IOG_L**	26, −86, −6		−5.42	178
**MOG_L**	26, −86, −6		−5.42	165
**IOG_R**	26, −86, −6		−5.42	106
**Cuneus_L**	26, −86, −6		−5.42	103
**Cuneus_R**	26, −86, −6		−5.42	103
**Fusiform_R**	26, −86, −6		−5.42	95
**PCG_L**	26, −86, −6		−5.42	84
**MOG_R**	26, −86, −6		−5.42	69
**MCG_R**	26, −86, −6		−5.42	56
**SOG_L**	26, −86, −6		−5.42	55
**PCG_R**	26, −86, −6		−5.42	51
**SOG_R**	26, −86, −6		−5.42	38
Female patients with LAT in CSF > Healthy subjects				
**Putamen_L**	−24, 6, −2	*p* = 0.001	5.40	507
**Caudate_L**	−24, 6, −2		5.40	23
**Amygdala_L**	−24, 6, −2		5.40	23
**Pallidum_L**	−24, 6, −2		5.40	20
Female patients with LAT in CSF < Healthy subjects				
**Precuneus_R**	10, −48, 50	*p* = 0.001	−4.10	190
Female patients with HAT in CSF > Female patients with LAT in CSF				
**STP_R**	46, 10, −18	*p* = 0.001	4.63	276
**MTP_R**	46, 10, −18		4.63	81
Female patients with HAT in CSF < Female patients with LAT in CSF				
**Calcarine_L**	12, −66, 26	*p* = 0.001	−4.94	122
**Precuneus_L**	12, −66, 26		−4.94	121
**Cuneus_L**	12, −66, 26		−4.94	90
**Calcarine_R**	12, −66, 26		−4.94	86
**Precuneus_R**	12, −66, 26		−4.94	36
Male patients with HAT in CSF > Healthy subjects				
**STG_L**	−28, 10, −4	*p* = 0.001	7.22	601
**Putamen_L**	−28, 10, −4		7.22	455
**Insula_L**	−28, 10, −4		7.22	256
**Amygdala_L**	−28, 10, −4		7.22	160
**STP_L**	−28, 10, −4		7.22	149
**Postcentral_L**	−28, 10, −4		7.22	115
**MTG_L**	−28, 10, −4		7.22	102
**RO_L**	−28, 10, −4		7.22	74
**Heschl_L**	−28, 10, −4		7.22	53
**MTP_L**	−28, 10, −4		7.22	50
**Hippocampus_L**	−28, 10, −4		7.22	38
**Supramarginal_L**	−28, 10, −4		7.22	38
**IOFG_L**	−28, 10, −4		7.22	27
Male patients with HAT in CSF < Healthy subjects				
**Precuneus_R**	10, −58, 26	*p* = 0.001	−5.22	168
**Calcarine_R**	10, −58, 26		−5.22	35
**Precuneus_L**	−6, −70, 46	*p* = 0.001	−5.06	194
Male patients with LAT in CSF > Healthy subjects				
**Putamen_L**	−52, −6, −4	*p* = 0.001	5.35	603
**STG_L**	−52, −6, −4		5.35	558
**Insula_L**	−52, −6, −4		5.35	447
**RO_L**	−52, −6, −4		5.35	148
**Pallidum_L**	−52, −6, −4		5.35	112
**STP_L**	−52, −6, −4		5.35	99
**Amygdala_L**	−52, −6, −4		5.35	78
**Caudate_L**	−52, −6, −4		5.35	76
**OIFG_L**	−52, −6, −4		5.35	73
**Postcentral_L**	−52, −6, −4		5.35	55
**Olfactory_L**	−52, −6, −4		5.35	44
**Heschl_L**	−52, −6, −4		5.35	39
**MTG_L**	−52, −6, −4		5.35	35
**Hippocampus_L**	−52, −6, −4		5.35	30
**MTP_L**	−52, −6, −4		5.35	28
Male patients with LAT in CSF < Healthy subjects				
**Precuneus_R**	18, −68, 40	*p* = 0.001	−6.40	527
**SOG_R**	18, −68, 40		−6.40	324
**Precuneus_L**	18, −68, 40		−6.40	307
**MOG_R**	18, −68, 40		−6.40	275
**SPG_R**	18, −68, 40		−6.40	270
**Cuneus_R**	18, −68, 40		−6.40	210
**Angular_R**	18, −68, 40		−6.40	180
**IPG_R**	18, −68, 40		−6.40	99
**MTG_R**	18, −68, 40		−6.40	66
**SPG_L**	18, −68, 40		−6.40	38
Female patients with HAT in CSF < Male patients with HAT in CSF				
**PCG_L**	−16, −48, 32	*p* = 0.001	−5.52	176
**Precuneus_L**	−16, −48, 32	*p* = 0.001	−5.52	125
**Calcarine_L**	−16, −48, 32	*p* = 0.001	−5.52	52
**MCG_L**	−16, −48, 32	*p* = 0.001	−5.52	45

CBF, cerebral blood flow; CSF, cerebrospinal fluid. MNI, Montreal Neurological Institute; FWE, Family-wise error; MTP, middle temporal pole; ITG, inferior temporal gyrus; MTG, middle temporal gyrus; MOFG, middle orbitofrontal gyrus; SOFG, superior orbitofrontal gyrus; IOFG, inferior orbitofrontal gyrus; TIFG, triangle inferior frontal gyrus; STP, superior temporal pole; OIFG, operculum inferior frontal gyrus; IOG, inferior occipital gyrus; SOG, superior occipital gyrus; MOG, middle occipital gyrus; STG, superior temporal gyrus; MTG, middle temporal gyrus; RO, rolandic operculum; SPG, superior parietal gyrus; IPG, inferior parietal gyrus; MCG, middle cingulum gyrus (MCG); PCG, post cingulum gyrus; L, left; R, right; HAT, high antibody titers; LAT, low antibody titers.

Compared with the healthy female subjects, the female patients with LAT in CSF showed an increase of CBF in the left putamen (Putamen_L), left caudate (Caudate_L), left amygdala (Amygdala_L), and left pallidum (Pallidum_L) (*p* = 0.001, cluster-level FWE corrected) (**Figure 1B**; **Table 2**). In contrast, the right Precuneus_R displayed decreased CBF (*p* = 0.001, cluster-level FWE corrected) (**Figure 1B**; **Table 2**).

Female patients with HAT in CSF showed more increased CBF in STP_R and MTP_R than those with LAT in CSF. CBF in Calcarine_L, Precuneus_L, Cuneus_L, Calcarine_R, and Precuneus_R in female patients with HAT in CSF was a greater decrease than that in the patients with LAT in CSF (*p* = 0.001, cluster-level FWE corrected) (**Figure 1C**; **Table 2**).

### 3.2 CBF chances in male patients

**Figure 2:**
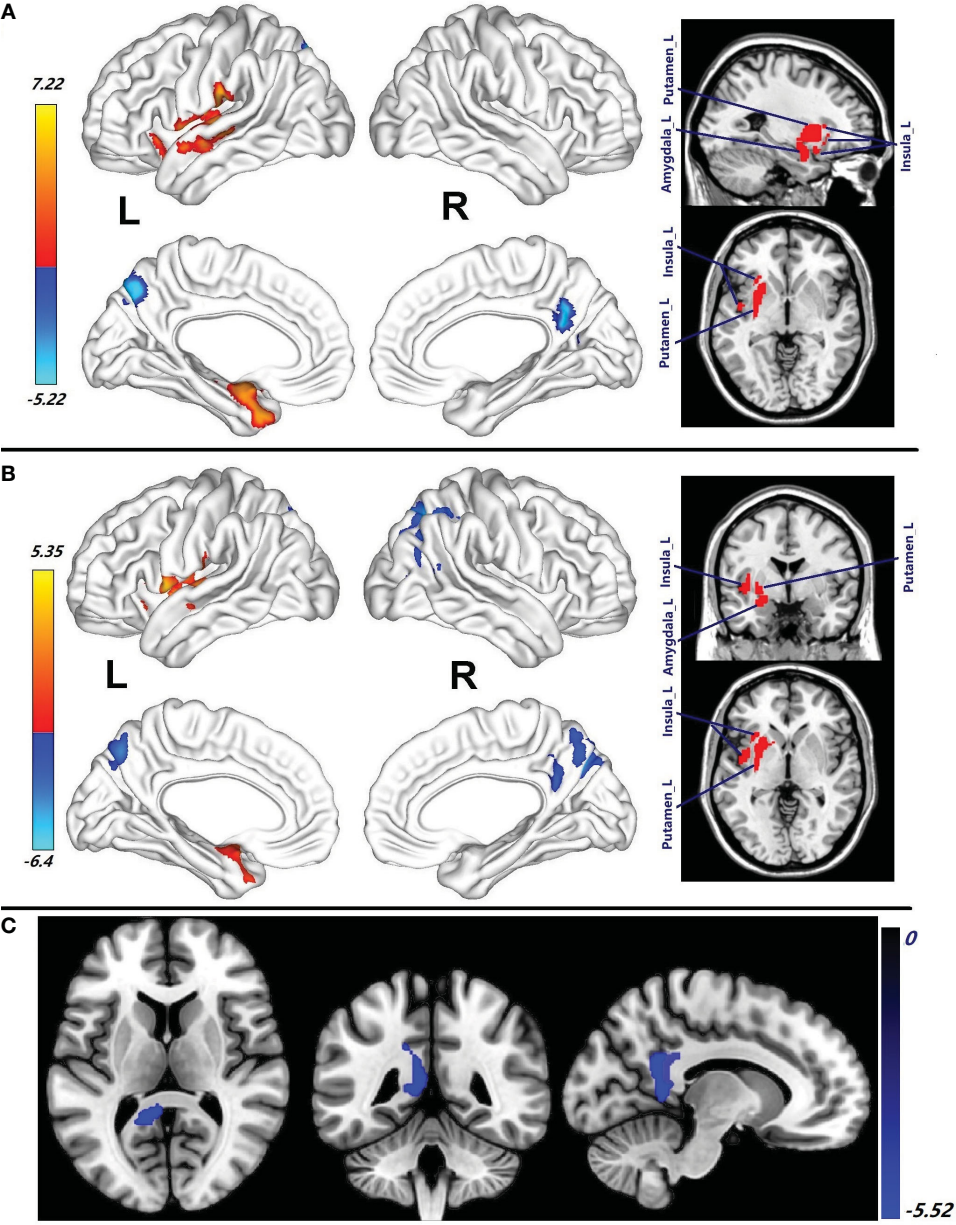
**(A)** Brain regions with CBF abnormalities between male patients with HAT in CSF and male healthy subjects (*p* = 0.001, cluster-level FWE corrected). **(B)** Compared with the healthy male subjects, the brain regions with CBF alteration in the male patients with LAT in CSF (*p* = 0.001, cluster-level FWE corrected). **(C)** Different CBF abnormalities between the female patients with HAT in CSF and the male patients with HAT in CSF (*p* = 0.001, cluster-level FWE corrected). Cool color: decrease in CBF; warm color: increase in CBF. HAT, high antibody titers; LAT, low antibody titers. NMDAR AE; anti-N-methyl-d-aspartate receptor autoimmune encephalitis.

Compared with the healthy male subjects, increase of CBF in the left superior temporal gyrus (STG_L), Putamen_L, left insula (Insula_L), Amygdala_L, left STP (STP_L), left postcentral (Postcentral_L), MTG_L, left rolandic operculum (RO_L), left heschl (Heschl_L), MTP_L, left hippocampus (Hippocampus_L), left supramarginal (Supramarginal_L), and IOFG_L was observed in male patients with HAT in CSF (*p* = 0.001, cluster-level FWE corrected) (**Figure 2A**; **Table 2**). In contrast, reduced CBF in the Precuneus_R and Calcarine_R was observed in male patients with HAT in CSF (*p* = 0.001, cluster-level FWE corrected) (**Figure 2A**; **Table 2**).

Compared with the healthy male subjects, the male patients with LAT in CSF showed increase of CBF in the Putamen_L, STG_L, Insula_L, RO_L, left pallidum (Pallidum_L), STP_L, Amygdala_L, Caudate_L, left OIFG (OIFG_L), Postcentral_L, Olfactory_L, Heschl_L, MTG_L, Hippocampus_L, and MTP_L (*p* = 0.001, cluster-level few corrected) (**Figure 2B**; **Table 2**). These patients, in contrast, exhibited significantly decreased CBF in Precuneus_R, right SOG (SOG_R), Precuneus_L, MOG_R, right (SPG_R), Cuneus_R, Angular_R, right inferior parietal gyrus (IPG_R), right MTG (MTG_R), and left superior parietal gyrus (SPG_L) (*p* = 0.001, cluster-level FWE corrected) (**Figure 2B**; **Table 2**).

There were no differences in CBF between male patients with HAT in CSF and male patients with LAT in CSF.

### 3.3 CBF differences between female patients and male patients

The decrease in CBF of PCG_L, Precuneus_L, Calcarine_L, and MCG_L observed in female patients with HAT in CSF was more pronounced compared to male patients with HAT in CSF (*p* = 0.001, cluster-level FWE corrected) (**Figure 2C**; **Table 2**). No differences in CBF were observed between female patients with LAT and the male patients with LAT in CSF.

### 3.4 Diagnostic CBF markers and connectivity in patients with NMDAR AE

**Figure 3:**
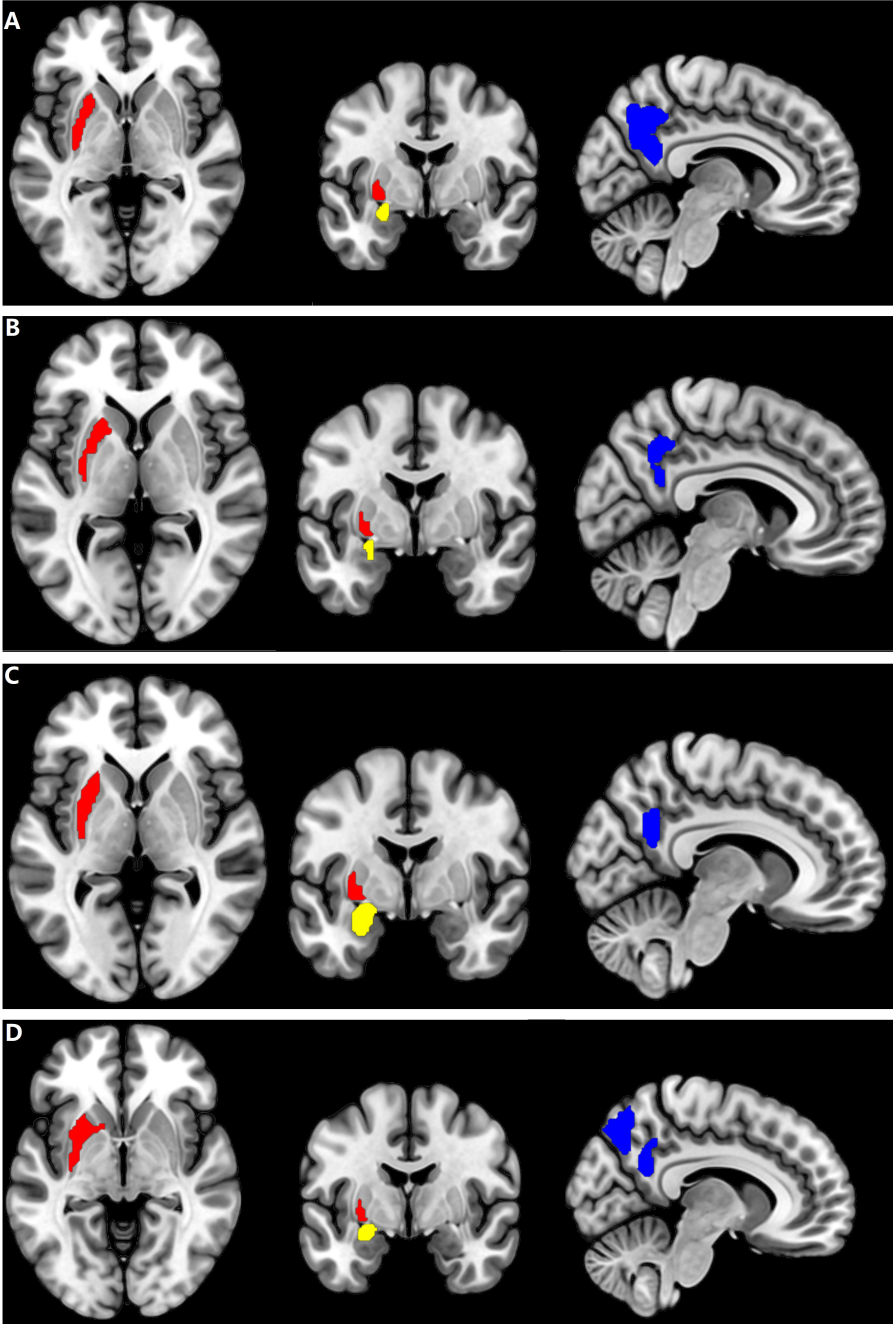
Brain regions with CBF alteration in all patients with different AT in CSF. **(A)** The Putamen_L, the Amygdala_L, and the Precuneus_R in female patients with HAT in CSF. **(B)** The Putamen_L, the Amygdala_L, and the Precuneus_R in female patients with LAT in CSF. **(C)** The Putamen_L, the Amygdala_L, and the Precuneus_R in male patients with HAT in CSF. **(D)** The Putamen_L, the Amygdala_L, and the Precuneus_R in male patients with LAT in CSF. Cool color: decrease of CBF in the Precuneus_R; red and yellow color: increase of CBF in the Putamen_L and Amygdala_L, respectively. AT, antibody titers; HAT, high antibody titers; LAT, low antibody titers.

**Figure 4:**
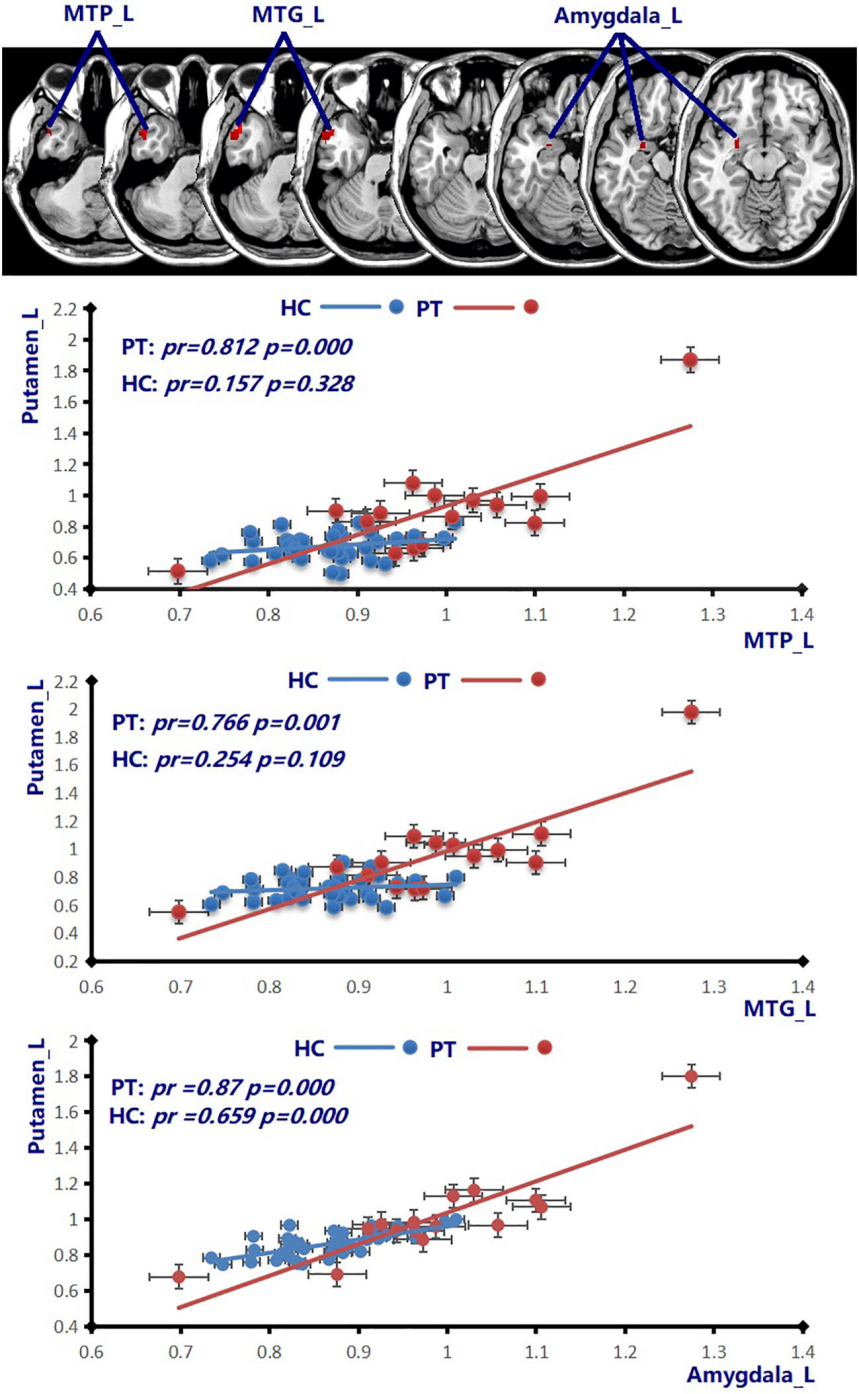
Compared to the healthy female subjects (blue line), the patients with HAT in CSF (red line) displayed increased positive CBF connectivity between the Putamen_L and MTP_L, MTG_L, and Amygdala_L (*p* = 0.001, cluster-level FWE corrected). Scatter plots illustrate the CBF connectivity in each group. MTP, middle temporal pole; MTG, middle temporal gyrus; HAT, high antibody titers.

The increase of CBF in the Putamen_L and Amygdala_L and decreased CBF in the Precuneus_R observed in all patients with NMDAR AE might serve as fundamental CBF markers (**Figure 3**). Compared with the healthy female subjects, the female patients with HAT in CSF displayed increased positive CBF connectivity between the Putamen_L and MTP_L, MTG_L, and Amygdala_L (*p* = 0.001, cluster-level FWE corrected) (**Figure 4**). Except for the female patients with LAT in CSF, the decrease of CBF in the Precuneus_L was observed in the remaining patients with NMDAR AE (*p* = 0.001, cluster-level FWE corrected) (**Table 2**).

## 4 Discussion

In addition to the 23 patients with NMDAR AE mentioned in our previous study (5), we successfully collected CBF data during the acute stage of the disease from an additional 22 patients for 4 years. The lack of specificity for conventional MRI abnormalities in patients with NMDAR AE makes it difficult for these data to contribute to the diagnosis of NMDAR AE (3, 18, 19). In our previous study on NMDAR AE, 60.9% of patients with normal MRI findings showed focal HCBF, and all patients with brain lesions displayed focal high blood flow (5). In the present study, 72.2% of patients with normal MRI findings showed focal HCBF. CBF abnormalities were more sensitive than conventional MRI abnormalities in patients with NMDAR AE (5). Decrease of NMDAR-mediated currents from reduced receptor density was associated with NMDAR AT (20–23). In our other recent studies, the clinical symptoms and EEG background activity were related to AT in CSF and sex (7, 10). The analysis of ROC curve and binary logistic regression revealed that CSF AT were significantly associated with unfavorable outcomes (7). Thus, CBF changes might also be related to AT in CSF and sex.

In this study, the pcASL technique was used to clarify increased CBF in the temporofrontal regions and subcortical nuclei, as well as decreased CBF in the parieto-occipital regions in patients with NMDAR AE (**Figures 1**, **2**; **Table 2**). According to the receptor maps of gene expression and protein density, the occipital regions and surrounding areas were high density for NMDAR, and the medial and lateral temporal and parietal association areas were both moderately dense for NMDAR (24). Furthermore, there was also a moderate expression of NMDAR in the basal ganglia (24). CSF NMDAR antibodies, which specifically bind to the GluN1 subunit of the NMDAR, led to internalization of the receptor, inhibition of ion entry by antibody blockade, and cell lysis mediated by complement (20, 25). NMDAR antibodies that target high-density NMDAR contributed to severely reduced neuronal activities (20–25), which might explain the decreased CBF in these regions in the precuneus and occipital regions (**Figures 1**, **2**; **Table 2**). The exact cause of the increased CBF remains uncertain. The inhibitory neurons exert intricate regulation over the firing of excitatory cells, owing to their extensive connectivity (21). NMDAR malfunction in inhibitory neurons and reduction of inhibitory synapses onto excitatory neurons were responsible for the decline in inhibition towards excitatory neurons in the temporofrontal regions and subcortical nuclei (21). Enhanced activities of excitatory neurons might lead to increased CBF in patients with NMDAR AE. Patients with different anti-NMDAR AT displayed different CBF abnormalities (**Figures 1**, **2**; **Table 2**). There were more brain regions with CBF abnormalities in female patients with HAT in CSF than in female patients with LAT in CSF (**Figures 1A, B**; **Table 2**). Furthermore, compared with female patients with LAT in CSF, more increased CBF abnormalities in the STP_R and MTP_R and more decreased abnormalities in the Calcarine_Bilateral, Precuneus_Bilateral, and Cuneus_L were observed in female patients with HAT in CSF (**Figure 1C**; **Table 2**). The EEG background activity during the acute stage in female patients with HAT in CSF exhibited greater severity compared to that observed in female patients with LAT in CSF, which is identical to the findings of our recent studies (7, 10). HAT in CSF contributed to more brains with CBF abnormalities, which might lead to more clinical symptoms and worse EEG background activity in female patients with HAT in CSF. In the present study, female patients with HAT in CSF experienced more decreased CBF abnormalities in the PCG_L, Precuneus_L, Calcarine_L, and MCG_L than the male patients with the same AT (**Figure 2C**; **Table 2**). The female patients experienced more clinical symptoms than the male patients, which was in accordance with our previous studies (7, 10). More decreased CBF in posterior brain regions might lead to worse EEG background activity. Female patients exhibited more severe EEG background activity at the peak stage compared to male patients (7, 10). In addition to CSF AT, sex also contributed to the altered CBF in patients with NMDAR AE.

If the same MRI abnormalities occur in patients with different AT in CSF simultaneously, the chances might be fundamental diagnostic biomarkers for the patients with NMDAR AE. In this study, increase of CBF in the Putamen_L and Amygdala_L and decreased CBF in the Precuneus_R in all patients with NMDAR AE might be CBF biomarkers contributing to the diagnosis of NMDAR AE (**Figure 3**; **Table 2**). Decreased Precuneus_L values were observed in all male patients and the female patients with HAT in CSF.

In a recent study recruiting 34 patients with NMDAR AE, all patients had a movement disorder (26). The putamen plays a crucial role in the processes of learning and motor control (27). The ventromedial neurons of the putamens are related to orofacial movements (27, 28). Increasing CBF abnormalities in the Putamen_L in all patients with NMDAR AE might contribute to movement disorders. Among various movement disorders, dystonia, chorea, and stereotypies were the principal dominant movement disorders (26). In our previous study, orofacial–lingual dyskinesia manifested in female individuals with HAT in CSF during the height of disease (7). Bilateral putamens were involved in female patients with HAT in CSF (**Figure 1A**; **Table 2**). In addition to the putamens, multiple basal regions with increased CBF abnormalities in patients with different AT in CSF might explain various movement disorders (**Figures 1**, **2**; **Table 2**). Increased positive CBF connectivity was observed among the Putamen_L and MTP_L, MTG_L, and amygdala_L in female patients with HAT (**Figure 4**). The basal ganglia regions not only participate in motor functions but also perform a vital role in complex goal-directed behaviors, which involve emotional processing, motivational drives, and cognitive aspects necessary for executing specific movements (27). The increased positive connectivity of CBF among the Putamen_L and MTP_L, MTG_L, and amygdala_L might contribute to the mental symptoms in the female patients with NMDAR AE.

Mental symptoms were the major symptom in patients with NMDAR AE. In this study, increased CBF in the temporal lobe in female patients with HAT in CSF (**Table 2**; **Figures 1**, **2**) might be associated with reductions in temporal interconnectivity (12). Decreased CBF in the precuneus was observed in all patients with NMDAR AE. The precuneus located in the medial and posterior regions of the parietal lobe is a pivotal node in the default mode network (DMN) (29). Except for maintaining the internal mental state in a state of rest, the precuneus participates in referential self-activity, storage of past experiences, sensory processing, emotional processing, spatial navigation, and future imagination (29, 30). The decreased CBF abnormalities in the precuneus might disturb the multimodal integration of the precuneus (31). Mental symptoms in patients with NMDAR AE might also resulted from alterations involving self-awareness and perception elaboration/integration in precuneus/DMN processing and the disruption of the networks (32–34). Eighty percent of female patients with HAT in CSF displayed mental symptoms as their initial symptoms. Only 20% of female patients with LAT in CSF manifested mental symptoms. More increased CBF abnormalities in the STP_R and MTP_R and more decreased CBF abnormalities in the Precuneus_Bilateral were observed in female patients with HAT in CSF than in the female patients with LAT in CSF (**Figure 1C**; **Table 2**). The insula plays a crucial role as a prominent constituent of the salience network (SN), which is responsible for identifying prominent stimuli and communicating with the DMN and central executive network (CEN) to conduct behaviors (35, 36). The increase of CBF in the insula might contribute to functional disruption (**Table 2**; **Figures 1**, **2**), which is in agreement with the findings that the decreased connectivity between the SN and the DMN/CEN is responsible for mental symptoms observed in schizophrenia (37). The amygdala, a key element of the limbic system, performs a crucial role in the initiation and spread of epileptiform activity among patients diagnosed with temporal lobe epilepsy (38). The activation of the amygdala during seizures elicits emotional symptoms, including fear, altered states of consciousness, memory flashbacks, and palpitations (39). The involvement of amygdala enlargement in autoimmune etiology is suggested by the observed reduction in seizure frequency and amygdala volume following immunotherapy (40). In the present study, increased CBF in the amygdala might contribute to seizure and emotional symptoms in patients with NMDAR AE.

The study encountered several limitations. First, the patient population is insufficient in size. During the acute stage of the disease, obtaining ASL data from certain patients with psychiatric and seizure symptoms poses challenges. Second, patients with NMDAR AE exhibited CBF changes across numerous brain regions in this study. Only Putamen_L, Amygdala_L, and the Precuneus_R were subjected to the CBF connectivity analysis. The analysis of CBF connectivity across the entire brain necessitates a data-driven approach.

## 5 Conclusion

Compared to the healthy subjects, the patients with different CSF AT exhibited different CBF abnormalities. Altered CBF in the Putamen_L, Amygdala_L, and Precuneus_R in all patients with NMDAR AE might be diagnostic CBF biomarkers for NMDAR AE. Female patients with HAT in CSF displayed more regions with CBF abnormalities than the female patients with LAT in CSF. More decreased CBF abnormalities in the PCG_L, Precuneus_L, Calcarine_L, and MCG_L were observed in the female patients with HAT in CSF than in the male patients with same AT. AT in CSF together with sex led to CBF changes in the patients with NMDAR AE.

## Data availability statement

The original contributions presented in the study are included in the article/supplementary material. Further inquiries can be directed to the corresponding author.

## Ethics statement

The study was approved by the ethical boards of the Affiliated Brain In review Hospital of Nanjing Medical University. The patients/guardians provided informed consent to participate in this study.

## Author contributions

AM: Writing – original draft. KW: Supervision, Writing – review & editing.
